# Low dose albumin for the prevention of renal impairment following large volume paracentesis in cirrhosis

**DOI:** 10.12669/pjms.313.7281

**Published:** 2015

**Authors:** Waqar Hussain, Abdullah Bin Khalid, Tayyab Usmani, Aiman Ghufran, Hasnain Shah

**Affiliations:** 1 Waqar Hussain, Section of Gastroenterology, Department of Medicine, Aga Khan University Hospital Karachi. Karachi Pakistan 74800; 2 Abdullah Bin Khalid, Section of Gastroenterology, Department of Medicine, Aga Khan University Hospital Karachi. Karachi Pakistan 74800; 3 Tayyab Usmani, Section of Gastroenterology, Department of Medicine, Aga Khan University Hospital Karachi. Karachi Pakistan 74800; 4 Aiman Ghufran, Section of Gastroenterology, Department of Medicine, Aga Khan University Hospital Karachi. Karachi Pakistan 74800; 5 Hasnain Shah, Section of Gastroenterology, Department of Medicine, Aga Khan University Hospital Karachi. Karachi Pakistan 74800

**Keywords:** Ascites, Child turcotte pugh score, Large volume paracentesis, Paracentesis induced circulatory dysfunction

## Abstract

**Objectives:**

To evaluate the effect of low dose Albumin i.e. 4 grams per litre of ascitic fluid after large volume paracentesis (LVP) for the prevention of paracentesis induced circulatory dysfunction (PICD) related renal impairment in cirrhosis.

**Methods:**

Case records of all patients with cirrhosis who underwent LVP from January 12^th^, 2011 till December 29^th^, 2013 were reviewed. Patients were excluded if they had spontaneous bacterial peritonitis, creatinine >1.5 mg/dl, hepatoma or if volume of ascitic fluid removed was <5 litres. Data including age, gender, cause of cirrhosis, CTP score and volume of ascitic fluid drained were noted. In addition serum creatinine and serum sodium at baseline and one week post paracentesis were recorded.

**Results:**

Two hundred and fourteen patients with cirrhosis underwent LVP during the study period. One hundred and thirty nine patients met the inclusion criteria and were analyzed. Patients were divided into two groups based on the amount of albumin given. The amount of albumin given was 25 grams and 50 grams while the volume of ascitic fluid removed were 6.2±1 litres and 10.4±1.5 litres in groups A and B respectively. One hundred and eight patients were in group A while thirty one patients were in group B respectively. Both groups received albumin at a dose of 4 grams per litre of ascitic fluid removed. Mean age in both groups were 53 years. Hepatitis C was the commonest etiology in both the groups, followed by Hepatitis B. More than 70% patients in both the groups were in child class C. Serum creatinine at baseline and one week post LVP was 1.04±0.24 mg/dl and 1.07±0.35 mg/dl in GROUP A while 1.11±0.23 mg/dl and 1.41±0.94 mg/dl in GROUP B. (P value 0.35). Similarly, serum sodium at baseline and one week post LVP was 130 ±5.6 meq/lit and 129.6±5.9 meq/lit in GROUP A while 127.6±5.8 meq/lit and 128±6.2 meq/lit in GROUP B respectively. (P value 0.14)

**Conclusion:**

This study suggests that 4 grams of albumin per litre of ascitic fluid drained is effective in preventing the PICD related renal impairment following large volume paracentesis in cirrhosis

## INTRODUCTION

Ascites is one of the most common complication of cirrhosis that leads to hospital admissions.^1^ The mainstay of ascites treatment is dietary sodium restriction and diuretics.^2^ However for ascites which is tense and in those patients where ascites is refractory to diuretic treatment, large volume paracentesis (LVP) of ascitic fluid provides rapid and effective relief of symptoms.^3^ The safety of performing serial LVP has been demonstrated and guidelines recommend serial LVP as one of the treatment options for refractory ascites.^4^

Removing large volume of ascitic fluid without volume expansion may lead to significant hemodynamic changes; a syndrome termed paracentesis induced circulatory dysfunction (PICD). PICD is associated with marked activation of renin angiotensin aldosterone system and is associated with renal impairment, re-accumulation of ascites and shortened survival.^5^ PICD can occur in upto 80% of patients not receiving plasma volume expansion. Various agents have been tried to prevent PICD during LVP. Amongst all of them albumin has been the most widely agent used so far and is found to be highly effective in preventing PICD following LVP.^6^-^8^ Albumin administration however has some potential side effects. Infusion of albumin markedly increase albumin degradation^9^ and albumin is prohibitively expensive.^10^-^12^ Moreover albumin administration post LVP does not offer a survival benefit as compared with those treated without albumin or with other plasma expanders. In prior studies, albumin has been administered at a dose of 6-8gm/litre of ascitic fluid removed.^5^,^13^ Only scarce literature exists comparing different doses of albumin, therefore American association of the study of liver diseases (AASLD) guidelines also state that albumin can be considered at a dose of 6-8 gm/litre of ascitic fluid removed.^4^

The aim of this study was to evaluate the effect of low dose albumin use for the prevention of PICD related renal impairment following large volume paracentesis in cirrhosis.

## METHODS

### Operational definitions

Renal failure is defined as >50% in the serum creatinine concentration to a value ≥1.5mg/dl from the baseline.^13^ Hyponatremia is defined as decrease in serum sodium to more than 5meq/litre to a level less than 130meq/litre.^13^

All patients of decompensated cirrhosis with tense ascites who underwent LVP between January 12^th^, 2011 till December 29^th^, 2013 at the section of gastroenterology, department of Medicine at the Aga Khan University Hospital were included in the study which was approved by the Aga Khan University Ethical Review Committee.

We included all patients with cirrhosis and tense ascites requiring LVP, age between 18 to 75 years who underwent paracentesis of >5 litres. The exclusion criteria were: Known coronary heart disease, congestive cardiac failure, creatinine more than 1.5mg/dl, spontaneous bacterial peritonitis, sepsis or variceal bleed within 7 days of LVP, hepatocellular carcinoma, platelet count less than 30,000 and malignant ascites.

The diagnosis of cirrhosis was made on clinical, biochemical and radiological features. A base line biochemistry panel including serum creatinine, serum sodium and potassium were obtained before paracentesis. Paracentesis was performed from right or left iliac fossa under sterile conditions. An albumin 4 gram per liter was administered immediately at the end of paracentesis. During and half an hour after paracentesis heart rate and blood pressures were monitored. After the administration of albumin the subjects were discharged and were asked to follow in the gastroenterology clinics after one week with biochemistry profile. On call physician assessed every patient for any local complications as well as for hemodynamic stability before discharge. At clinic visit, patient’s serum creatinine and sodium were compared with pre paracentesis profile.

### Statistical Method

Results were expressed as mean + standard deviation for continuous variables (e.g., Age) and number (percentage) for categorical data (e.g. Gender etc.). Groups were compared using the independent sample t-test, Pearson Chi-square test and Fisher Exact test where ever appropriate. A P-value of < 0.05 was considered as statistically significant. All p-value were two sided. Statistical interpretation of data was performed by using the computerized software program SPSS version 19.0.

## RESULTS

Two hundred and fourteen patients underwent LVP during the study period. Seventy five patients were excluded out of which eleven had malignant ascites, forty three had serum creatinine >1.5 mg/dl. The data was incomplete for twenty one patients. Total One hundred and thirty nine patients were analyzed. (Fig.1). They were divided in two groups on the basis of amount of albumin given. The amount of albumin given was 25 grams and 50 grams while the volume of ascitic fluid removed were 6.2±1 and 10.4±1.5 in groups A and B respectively. One hundred and eight patients were in group A while thirty one patients were in group B respectively. Both groups received albumin at a dose of 4 grams per litre of ascitic fluid removed. Mean age was 53±12.5 in the group A while 52.8±10.1 in group B. hepatitis C was the predominant etiology in both the groups, seventy six patients (70.4%) in group A while seventeen patients (54.9%) in group B followed by hepatitis B, seventeen patients (15.7%) in group A and six patients (19.3%) in group B. Majority of patients were in Child Turcotte Pugh score of class C, 76% and 71% in group A and group B respectively. (Table-I)

**Figure:**
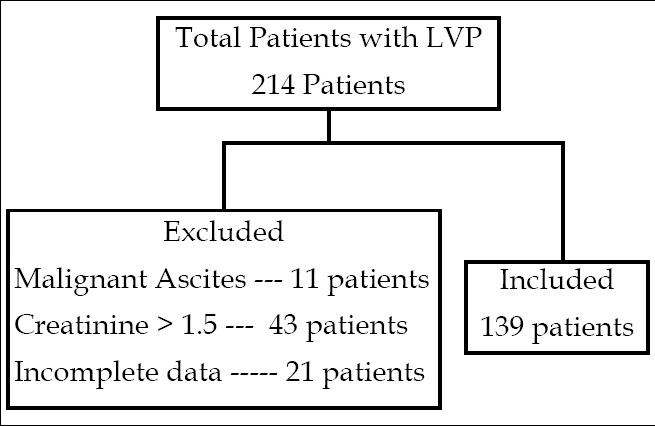


**Table-I:** Shows the baseline characteristics of the two groups.

	Group A (Albumin 25gm) (n=108)	Group B (Albumin 50gm) (n=31)	P value
Age(yrs)	53.2 ± 11.2	52.8± 10.1	0.47
Gender (M/F)	58/50	15/16	0.68
*Etiology*			
Hepatitis C	76(70.4%)	17(54.9%)	----
Hepatitis B	17(15.7%)	6(19.3%)	
Non B Non C	15(13.9%)	8(25.8%)	
T.Bilirubin (mg/dl)	4.5± 5.0	4.9 ± 5.8	0.69
Albumin (g/l)	2.16± 0.42	2.29± 0.51	0.13
Prothrombin time	18.4 ± 5.08	19.36 ± 5.79	0.08
Child score	11 ± 2	11±2	0.24
*Child Class*			
B	26(24.1%)	9(29%)	0.64
C	82(75.9%)	22(71%)	
Creatinine (mg/dl)	1.04± 0.24	1.11± 0.23	0.15
Sodium (meq/lit)	130 ± 5.6	127.6 ± 5.8	0.01
Volume of ascitic fluid drained	6.2± 1	10.4±1.5	––

There was a rise in serum creatinine 6-10 days post LVP in group B from 1.11 ± 0.23 to 1.41±0.94 as compared to the group A 1.04 ± 0.24 pre LVP to 1.07 ± 0.35 post LVP; but the difference did not reach statistical significance (*P* value 0.35). Similarly the difference in serum sodium level from baseline to 6-10 days post LVP also did not reach statistical significance. (Table-II)

**Table-II:** Changes in serum creatinine and sodium pre and post LVP.

	Group A (25gram Albumin) (n=108)		Group B (50gram Albumin) (n=31)		P value
	Pre LVP	Post LVP	Pre LVP	Post LVP	
Serum Cr (mg/dl)	1.04± 0.24	1.07±0.35	1.11± 0.23	1.41± 0.94	0.35
Sodium (meq/lit)	130 ± 5.6	129.6±5.9	127.6 ± 5.8	128 ±6.2	0.14

Five (4.62%) patients in the group A while Two (6.45%) patients in the group B had an increase of serum creatinine level of >0.5 mg/dl. Similarly, hyponatremia i.e. serum sodium <5 meq/lit from the baseline was observed in nine patients (8.33%) in the group A while three patients (9.67%) in the group B respectively. Three patients (2.3%) in group A and one patient (3.22%) in group B had transient hypotension which resolved by transiently stopping the ascitic drainage. None of the patients had abdominal wall hematoma or any other complication during the procedure.

## DISCUSSION

LVP in cirrhotic patients with ascites without volume expansion leads to complex circulatory abnormalities termed PICD which is clinically manifested by renal impairment, reaccumulation of ascites and shortened survival.^14^ In the initial phase post LVP there is an increase in cardiac output and stroke volume accompanied by a deactivation of the renin angiotensisn and sympathetic nervous system. This early phase is followed by a late phase in which there is an increase in the sympathetic and vasoconstrictor systems. It was also observed that these neurohumoral changes are associated with a decrease in systemic vascular resistance led to the conclusion that an accentuation of vasodilation is responsible for PICD in cirrhotic patients.^6^-^8^ This circulatory disturbance post LVP not only causes the hemodynamic alterations but is also responsible for worsening renal function, dilutional hyponatremia, increased accumulation of ascites and decreased survival.^15^ Various agents have been tried post LVP albeit with variable success to prevent PICD related complications mainly the renal impairment. Amongst those albumins has been found to be most effective.^16^-^18^

Trials have also shown that when paracentesis of less than 6 litres is performed, the incidence of complications is the same irrespective of the plasma expander used but when LVP of more than or equal to 6 liters is performed, Albumin is superior as compared to other plasma expanders.^5^,^19^,^20^ However albumin is very expensive. Initial studies done on this aspect used albumin at a dose of 8 gram/liter. There is only scarce data so far that compared albumin at a lower dose than conventionally used.

In our study we did not find any statistically significant difference in the frequency of renal dysfunction and hyponatremia in the two groups at a dose of 4 grams albumin per litre of ascitic fluid drained. By definition, renal impairment occurred in five patients in group A and two patients in group B which were statistically not significant. Prior studies using a higher dose of albumin showed a similar statistically insignificant difference of renal dysfunction post LVP.^21^

This finding has important bearing both from clinical and financial point of view. If this point is further proven in prospective randomized clinical trials it means that these patients can easily be managed with a lower dose of albumin without putting them at increased risk of renal failure.

Serum creatinine is also a part of the model for end stage liver disease (MELD) score which predicts short term mortality in a decompensated cirrhotic patient. A higher serum creatinine implies a high MELD and vice versa. Moreover studies have shown that even after the post transplantation incidence of renal dysfunction depends on pretransplant kidney function.^22^ Hence, stabilization of renal function is very important in cirrhotic patients. The integral part in diagnosing PICD is increase in serum renin levels observed 5-6 days after paracentesis; since our study was retrospective we could not obtain serum renin levels in our patients. However the clinical manifestation of PICD is renal dysfunction and hyponatremia, which were well captured in our study. We segregated our patients into two groups based on the amount of albumin given. Since this was a retrospective study the two groups were not equally divided in number of patients. It is well known that if a larger volume of ascites is removed the greater is the chance of PICD and subsequent renal dysfunction.^5^ It is however, interesting to note that group B in our study in which the mean amount of ascites drained was 10 liters the rate of renal dysfunction was the same as in group A in which lesser amount of ascitic fluid was drained. Similarly, hyponatremia i.e. serum sodium <5 meq/lit from the baseline was observed in nine patients (8.33%) in the group A while three patients (9.67%) in the group B respectively which is consistent with the previous study done by Carlo Alessandria *et al*.^21^

## CONCLUSION

The results of this study suggested that treatment with low dose albumin i.e. 4 grams per litre of ascitic fluid drained is effective and safe in the prevention of renal impairment after large volume paracentesis in cirrhotic patients with tense ascites. However, any final recommendation regarding the exact dosage of albumin will require prospective, randomized control trials incorporating serum renin as an outcome parameter along with renal impairment and hyponatremia.
